# Delineation of molecular subtypes based on pathway activation signatures reveals intrinsic tumor heterogeneity and pan-cancer shared features

**DOI:** 10.3389/fonc.2026.1918117

**Published:** 2026-09-16

**Authors:** Ren Xu, Xiao Xiao, Jinglin Yang, Tingting Wang, Yuanhua Yu, Hao Zhang

**Affiliations:** 1 School of Physics, Changchun University of Science and Technology, Changchun, China; 2 School of Life Science and Technology, Changchun University of Science and Technology, Changchun, China

**Keywords:** drug sensitivity, heterogeneity, molecular subtypes, pan-cancer, pathway activity signatures

## Abstract

**Purposes:**

Individual molecular features are insufficient to elucidate the complex biological properties of pan-cancer. Accordingly, this study performed pan-cancer molecular subtyping based on pathway activation signatures to dissect the heterogeneity and commonality across malignancies.

**Methods:**

Transcriptomic data for 7,138 pan-cancer samples were retrieved from TCGA database for molecular subtyping. In parallel, transcriptomic data for 597 cell lines and drug sensitivity data for 269 lines were obtained from CCLE database for drug sensitivity analysis, and transcriptomic profiles of 16 in-house cell lines were incorporated for combined analysis.

**Results:**

Using unsupervised clustering, we classified pan-cancer into four distinct molecular subtypes based on the activity of 43 key cancer-related pathways. C1 was enriched for upregulated pathways involved in oncogenic signaling, cellular processes-related and immune pathways. C2 was characterized by upregulated activation of metabolic and DNA damage response (DDR) pathways. C3 displayed upregulated activity of metabolic, DDR and immune pathways. C4 showed elevated activity of oncogenic signaling, cellular processes-related, and DDR pathways. Notably, compared with C2 and C4, C1 and C3 displayed enhanced immune score, increased CD8+T cell infiltration and higher expression of genes involving in antigen processing and presentation, immunogenic cell death and immune checkpoint. C3 demonstrated the most favorable overall survival and the lowest proportion of progressive/stable disease patients, followed by C1, whereas C2 and C4 were associated with inferior clinical outcomes. C3 was susceptible to multiple agents, while C1 showed multidrug resistance. Moreover, *in vitro* cellular experiments demonstrated that combined treatment with inhibitors targeting activated pathways yielded superior inhibitory effects relative to monotherapy for C1, C2 and C4.

**Conclusions:**

Overall, our study defined four molecular subtypes based on the pathway activation profiles, with each subtype displaying unique features in pathway activation, clinical manifestations, treatment response, survival prognosis, immune microenvironment, and drug sensitivity. These findings may provide potential valuable insights for the precise diagnosis and treatment of cancer.

## 1 Introduction

Cancer represents one of the leading causes of death globally, accounting for approximately 20 million new cases and 10 million cancer-related deaths annually (1, 2). The ever-escalating burden of cancer incidence and mortality constitutes a critical public health challenge, driving a substantial surge of research dedicated to the pathogenesis, prevention, and clinical management of cancer. Notably, cancer is a highly heterogeneous disease, with significant molecular mechanistic differences often observed even among tumors of the same tissue type (3, 4). Meanwhile, mounting evidence suggests that some tumors across multiple tissues may share similar characteristics, such as common etiology, and therapeutic targets (5, 6). Pan-cancer analysis helps reveal the heterogeneities and commonalities across different cancers at the clinical, genomic, epigenomic, transcriptomic, and proteomic levels (7, 8). To date, multiple pan-cancer studies have identified a set of molecular biomarkers for prognostic assessment and therapeutic guidance in cancer patients (9, 10). For example, Gammall et al. (2) identified 440 gene mutations associated with survival prognosis in cancer patients, covering key genes including *TP53*, *BRCA1*, *BRCA2*, *BRAF*, and *KRAS*. Food and Drug Administration (FDA) has approved multiple biomarker-guided therapies covering targets such as *NTRK* fusions and *BRAF* V600E mutations (11, 12). Although an increasing number of cancer biomarkers have been identified, individual biomarker are rarely sufficient to account for the heterogeneity in clinical outcomes and drug response among patients (13, 14). For instance, *EGFR* mutation status is an FDA-approved marker for erlotinib response in non-small cell lung cancer (NSCLC), yet clinical data show a response rate of only 16% in *EGFR*-mutant tumors, indicating that other confounding factors drive drug resistance (15). Notably, a growing body of studies have indicated that cancer is essentially driven by dysregulation of complex regulatory relationships among multiple functionally relevant genes (16). These observations suggest that tumor should be interpreted at the level of multiple functional modules (e.g., biological pathways), rather than individual genes or molecules (17, 18). However, researches integrating multiple distinct pathways for elucidating the heterogeneity and commonality of pan-cancer remain rare.

Therefore, this study aimed to construct a pan-cancer molecular subtyping system based on the activity of multiple key cancer-related signaling pathways. To this end, we first performed pan-cancer molecular subtyping based on the activity profiles of multiple canonical cancer pathways across The Cancer Genome Atlas (TCGA) specimens. Subsequently, we compared the differences of clinical characteristics, treatment response, prognostic outcomes, and tumor immune microenvironment (TIME) across distinct subtypes. Then, we developed a machine learning model to predict the defined subtypes to facilitate the clinical translation of this subtype system. Finally, by integrating cancer cell line datasets and performing subtype annotation using the cell line-specific prediction model, we compared the drug sensitivity profiles among different subtypes. The workflow of this study is illustrated in **Figure 1**.

**Figure 1:**
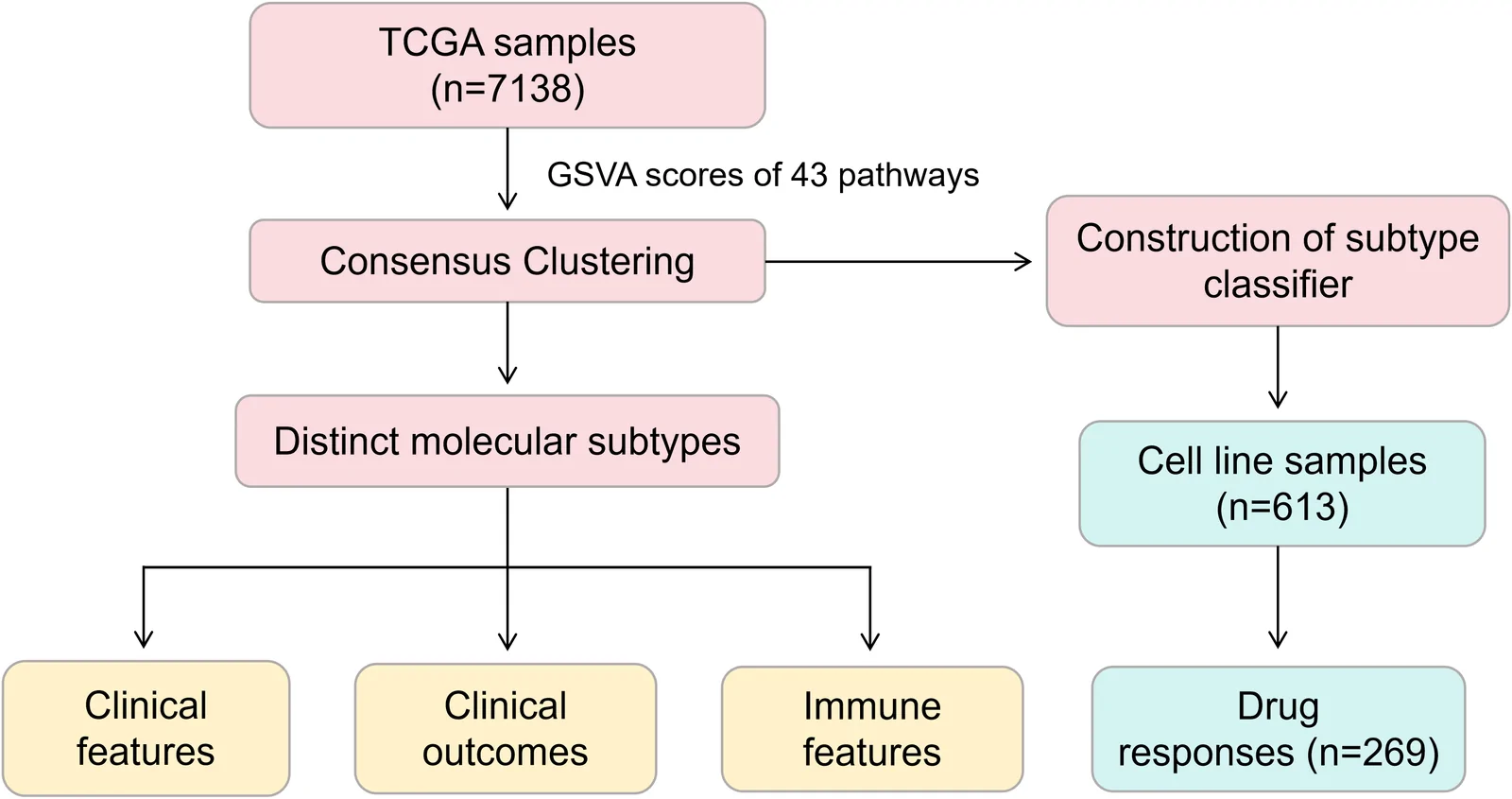
The flowchart of this study.

## 2 Method

### 2.1 Data acquisition

Gene expression profiles, and clinical phenotypic data of cancer patients used in this study were downloaded from TCGA database (https://portal.gdc.cancer.gov). The final available data comprised 7138 tumor samples from 20 tumor types, including breast invasive carcinoma (BRCA, n=1095), kidney renal clear cell carcinoma (KIRC, n=533), head and neck squamous cell carcinoma (HNSC, n=520), lung adenocarcinoma (LUAD, n=515), lung squamous cell carcinoma (LUSC, n=501), thyroid carcinoma (THCA, n=505), prostate adenocarcinoma (PRAD, n=497), stomach adenocarcinoma (STAD, n=415), bladder urothelial carcinoma (BLCA, n=407), liver hepatocellular carcinoma (LIHC, n=371), ovarian serous cystadenocarcinoma (OV, n=305), cervical squamous cell carcinoma and endocervical adenocarcinoma (CESC, n=302), colon adenocarcinoma (COAD, n=283), esophageal carcinoma (ESCA, n=184), pancreatic adenocarcinoma (PAAD, n=178), uterine corpus endometrial carcinoma (UCEC, n=177), acute myeloid leukemia (LAML, n=173), rectum adenocarcinoma (READ, n=93), lymphoid neoplasm diffuse large B-cell lymphoma (DLBC, n=48), cholangiocarcinoma (CHOL, n=36, **Supplementary Table 1**). In addition, we obtained gene expression profiles and drug sensitivity data of corresponding pan-cancer cell lines from the Cancer Cell Line Encyclopedia (CCLE) database (https://sites.broadinstitute.org/ccle/). Meanwhile, we performed RNA sequencing (RNA-seq) on 16 cancer cell lines in our laboratory, and integrated the in-house sequencing data with the public datasets for comprehensive joint analysis. The cell lines present in this study were obtained from Shanghai Institute for Biological Sciences, Chinese Academy of Sciences. To mitigate batch effects arising from different cohorts, batch-effect correction for transcriptomic data was performed using the ComBat algorithm in the R package sva (v3.42.0). Principal component analysis (PCA) was performed to assess batch effect removal efficiency.

### 2.2 Consensus clustering

This study performed molecular classification of 7,138 samples from TCGA database based on the pathway activity profiles of 43 key functional pathways, including oncogenic signaling pathways, immune pathways, metabolic pathways, DNA damage response (DDR) pathways, as well as pathways associated with cellular processes, transport and protein degradation, and drug resistance (**Supplementary Table 2**). The gene sets corresponding to each pathway were retrieved using the R package KEGGREST (v1.46.0). Subsequently, the Gene Set Variation Analysis (GSVA) R package (v2.0.7) was applied to calculate the pathway activity scores of individual samples. Based on these GSVA scores, ConsensusClusterPlus package (v1.70.0) was used for unsupervised clustering to identify distinct molecular subtypes. Samples were classified into clusters by using the Pearson distance metric and the partitioning around medoids (PAM) algorithm with setting from 2 to 10. Each bootstrap contained around 80% of the samples, compiling the results for 50 resampling repetitions. The optimal value for the clustering number was determined by analyzing the consensus matrix heatmap, cumulative distribution function (CDF) plot and delta area plot.

### 2.3 Assessment of immune microenvironment based on molecular cluster

ESTIMATE package (v 1.0.13) was used to evaluate the immune cell infiltration level (immune score), stromal content (stromal score), tumor purity, and composite ESTIMATE score for each sample. CIBERSORT algorithm was applied to assess the abundance of 22 types of immune cells in individual samples. The expression levels of key immune-related genes among distinct molecular subtypes were compared using Kruskal-Wallis tests.

### 2.4 Construction of a classification model based on pathway heterogeneity

To improve the clinical translational potential of molecular subtyping, we constructed a predictive model using the Elastic net algorithm. Samples from the TCGA database were randomly divided into training and validation set at a ratio of 7:3. Using the R package caret (v7.0.1), the molecular subtyping model was constructed in the training set with GSVA scores of 43 pathways as input features. The tuning parameters alpha and lambda values were optimized by 10-fold cross-validation to maximize classification accuracy. In the training set, the sensitivity, specificity, and accuracy were used to evaluate the classification power of the model. Then, this model was validated in the validation set.

### 2.5 Drug response analysis based on molecular cluster

To uncover the differential drug response across different molecular cluster, we downloaded *in vitro* drug response data [Drug sensitivity AUC (Sanger GDSC2)] from CCLE database. Considering the inherent biological heterogeneity between cell-line models and clinical tumor tissues, we implemented rigorous subtype assignment thresholds to minimize classification bias. Based on the predictive model built from TCGA clinical samples, only cell lines with a subtype prediction probability exceeding 0.9 were assigned to the corresponding subtype. Moreover, only drugs with at least two drug-sensitivity observations per molecular subtype were retained for downstream analysis, yielding a final cohort of 169 compounds. Two-sided Wilcoxon rank-sum tests were used to compare AUC levels of different drugs between a given subtype and all remaining subtypes. Raw *P* values derived from these tests were adjusted via the Benjamini-Hochberg false-discovery rate (FDR) procedure to correct for multiple hypothesis testing across multiple comparisons. We considered cluster sensitive to a drug if the adjusted *P* < 0.05 and the scaled AUC<0 in this cluster, and cluster resistant to a drug if the adjusted *P* < 0.05 and the scaled AUC>0 in this cluster.

### 2.6 Drug sensitivity assay

Finally, we experimentally validated the hypothesized benefits of combination drug in C1, C2, and C4 using cell lines. For each molecular subtype, we prioritized cell lines that were available in our laboratory and had a high predicted probability for that subtype. Three selected cell lines corresponding to each subtype-T24 (urothelial bladder carcinoma), MDA-MB-453 (hormone receptor-positive breast cancer), and BT20 (triple-negative breast cancer) were previously purchased from the Shanghai Institute for Biological Sciences, Chinese Academy of Sciences. These cell lines were cultivated in Dulbecco’s modified Eagles medium (DMEM) (Gibico, China), supplemented with 10% fetal bovine serum (FBS) and 1% penicillin-streptomycin-amphotericin B solution at 37 °C in a 5% CO2 atmosphere. Cells at the logarithmic growth phase were trypsinized to generate a single-cell suspension and seeded into 96-well plates at a density of 5×10^3^ cells per well. Then, T24 cells were incubated with either DAPT (NOTCH pathway inhibitor) alone, or tariquidar (ABC transporter inhibitor) alone, or a mixture of both for 72 h; MDA-MB-453 were incubated with either 2-Deoxy-D-glucose (2-DG, glycolysis inhibitors) alone, or cytarabine (DDR pathway inhibitor) alone, or a mixture of both for 48 h; BT20 were incubated with either DAPT alone, or cytarabine alone or a mixture of both for 72 h. The concentrations of all compounds and the incubation times were determined in preliminary experiments. Subsequently, 10 μL Cell Counting Kit-8 (CCK-8, biosharp, China) was added and culture was continued for 1 h. Absorbances at 450 nm were measured by Tecan Infinite^®^ F50 microplate reader (Tecan Group Ltd., Switzerland). All assays were carried out independently in triplicate.

### 2.7 Statistical analysis

Statistical analysis was performed using R package (version 4.1.2). Categorical variables were compared using Fisher’s exact test or Chi-square test. Quantitative data was compared by Kruskal-Wallis tests or two-sided Wilcoxon rank-sun tests. Overall survival (OS) was defined as the time from the diagnosis to the last follow-up or death due to any cause. Kaplan-Meier method with log-rank test was used to estimate OS. All tests were two-tailed, with statistical significance denoted as *P < 0.05, **P < 0.01, and ***P < 0.001. Graphs were made in Adobe Illustrator 2021 (Adobe Inc.).

## 3 Results

### 3.1 Unsupervised hierarchical clustering revealed distinctive pathway activation signatures in pan-cancer

Based on the activity levels of 43 key pathways, we performed consensus clustering analysis on pan-cancer samples to delineate distinct pathway activation signatures across cancers. After batch effect adjustment, batch-related discrepancy among TCGA samples was effectively removed (**Supplementary Figures 1A, B**). As illustrated in **Figures 2A, B**, the optimal number of clusters was determined to be k = 4 since the delta area and consensus CDF increased slowly after k = 4. Moreover, the consensus matrix at k = 4 displayed clear diagonal block structures, high intra-cluster consistency and distinct inter-cluster boundaries, demonstrating stable clustering performance. In contrast, when k ≥ 5, the consensus matrices yielded fragmented block structures, blurred subtype demarcations and ambiguous sample assignments, which greatly degraded clustering stability (**Supplementary Figure 1C**). These results further supported k = 4 as the optimal clustering solution. Notably, the four molecular subtypes had distinct heterogeneity in pathway activation patterns (**Figure 2D**). Specifically, C1 (n=2775, 38.9%) was characterized by upregulation of oncogenic signaling pathways, immune pathways, cellular processes-related pathways, as well as ABC transporter pathway. C2 (n=1712, 24.0%) showed prominent activation of metabolic pathways and DDR pathways. C3 (n=1634, 22.9%) displayed high activation of immune pathways, metabolic pathways, and DDR pathways. C4 (n=1017, 14.2%) presented elevated activation of oncogenic signaling pathways, cellular processes-related pathways, as well as DDR pathways.

**Figure 2:**
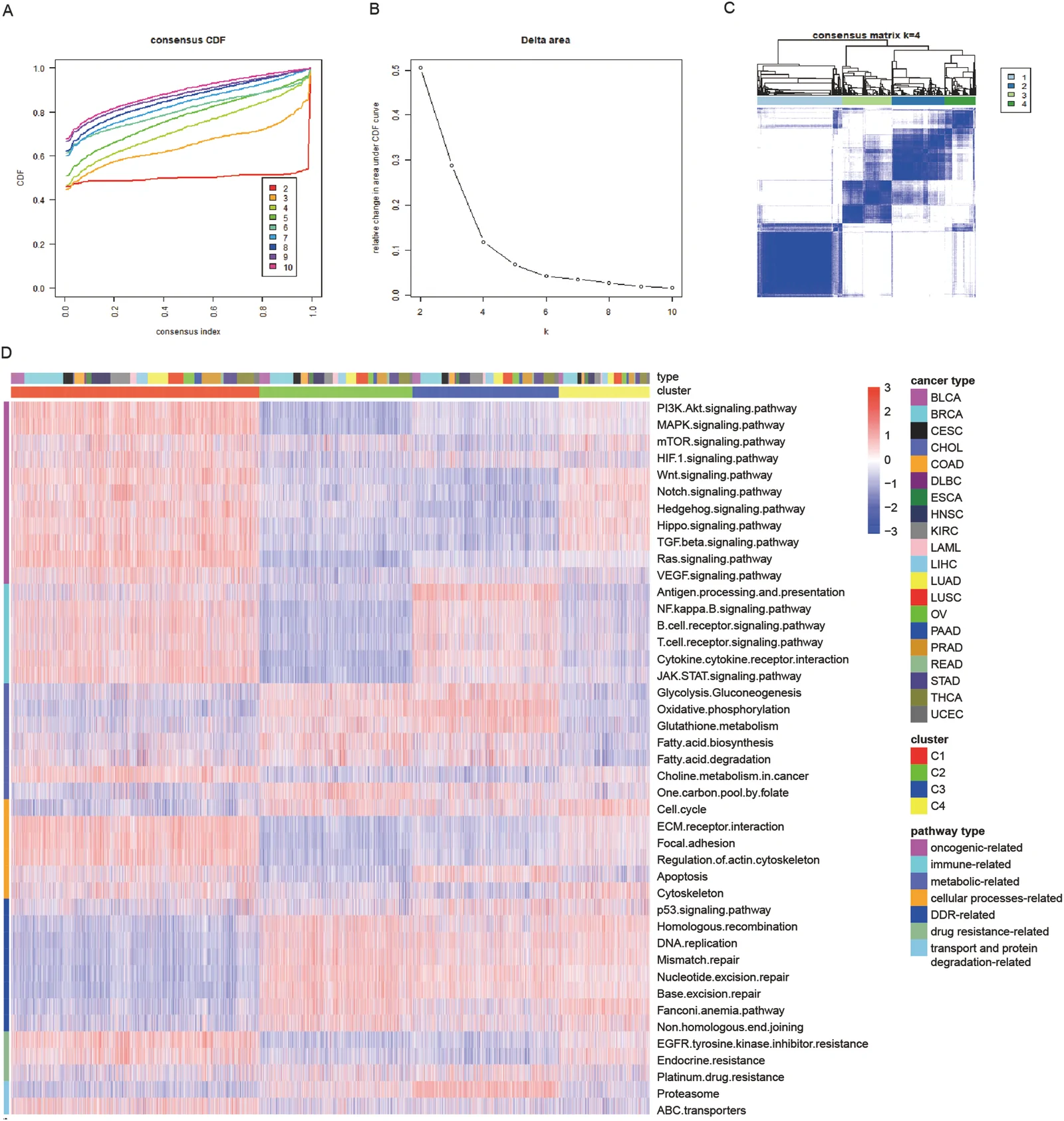
Construction of four molecular subtypes through consensus clustering based on the activity of 43 pathways. **(A)** The cumulative distribution function (CDF) curves for k=2 to k=10. **(B)** Relative change in area under the CDF curve for k=2 to k=10. **(C)** The consensus matrix obtained when k=4. **(D)** Heatmap showing the activity of 43 pathways among the four molecular subtypes in the TCGA cohort.

Subsequently, we compared the differences in clinical characteristics, therapeutic efficacy and survival outcomes across the four subtypes (**Table 1**). The distribution patterns of various subtypes across cancer types were highly consistent, with C1 representing the most prevalent subtype in all cancers. Moreover, apart from CHOL and UCEC, patients classified into the C4 accounted for the lowest proportion across all other cancer types (**Figure 3A**). These distribution patterns indicated that this molecular classification may be broadly applicable to various individual cancer types. Moreover, patients in C4 were significantly younger than those in the other subgroups (*P* < 0.05, **Figure 3B**). C1 and C3 had a markedly higher proportion of female patients (*P* = 0.002, **Figure 3C**), and C1 had a relatively higher proportion of white patients (*P* < 0.001, **Figure 3D**). Such discrepancies in demographic distribution may stem from the absence of stratified balancing screening for samples of diverse tumor types included in this study. C4 presented a remarkably higher proportion of patients with squamous cell carcinoma (*P* = 0.05, **Figure 3E**). No significant differences were observed regarding tumor stage across different subtypes (*P* = 0.93, **Figure 3F**). Albeit not statistically significant, C2 and C4 showed a notably higher proportion of patients with progressive disease (PD) or stable disease (SD, *P* = 0.052, **Figure 3G**). Furthermore, the median survival times for C1, C2, C3, and C4 were 84.4, 82.3, 98.8, and 69.4 months, respectively. The Kaplan-Meier curve demonstrated significant survival differences among the four subtypes (*P* = 0.016, **Figure 3H**); however, pairwise comparisons revealed significant survival disparities only between certain subgroups (C1 vs. C4, *P* = 0.039; C2 vs. C3, *P* = 0.017; C3 vs. C4, *P* = 0.004; C1 vs. C2, *P* = 0.232; C1 vs. C3, *P* = 0.145; C2 vs. C4, *P* = 0.372). C3 exhibited the most favorable prognosis, followed by C1, whereas C2 and C4 was associated with the worst outcome. Using C3 as the reference group, the hazard ratio for C1, C2 and C4 was 1.088 (95%CI, 0.970-1.221), 1.165 (95%CI, 1.027-1.321), and 1.243 (95%CI, 1.075-1.437).

**Table 1:** Baseline characteristics of patients in the TCGA cohort.

Characteristic	Overall (n=7138)	C1 (n=2775)	C2 (n=1712)	C3 (n=1634)	C4 (n=1017)	*P* value
Age [years, median (ranges)]						
Gender [n, (%)]						0.002
Female	3742 (52.4)	1520 (54.8)	847 (49.5)	867 (53.1)	508 (50.0)	
Male	3396 (47.6)	1255 (45.2)	865 (50.5)	767 (46.9)	509 (50.0)	
Race [n, (%)]						<0.001
American Indian or Alaska native	18 (0.3)	7 (0.3)	4 (0.3)	5 (0.4)	2 (0.2)	
Asian	564 (9.1)	176 (7.1)	160 (10.9)	134 (9.7)	94 (10.6)	
Black or African American	663 (10.7)	227 (9.2)	192 (13.1)	156 (11.3)	88 (9.9)	
Native Hawaiian or other pacific islander	7 (0.1)	2 (0.1)	1 (0.1)	3 (0.2)	1 (0.1)	
White	4950 (79.8)	2059 (83.3)	1109 (75.6)	1080 (78.4)	702 (79.1)	
Histological type [n, (%)]						0.05
Adenocarcinoma	2573 (65.3)	1029 (66.8)	619 (65.9)	583 (65.4)	342 (60.3)	
Squamous cell carcinoma	1366 (34.7)	512 (33.2)	321 (34.1)	308 (34.6)	225 (39.7)	
Stage [n, (%)]						0.93
Stage I/II	3416 (63.0)	1338 (63.4)	814 (62.7)	780 (63.0)	484 (62.2)	
Stage III/V	2008 (37.0)	771 (36.6)	485 (37.3)	458 (37.0)	294 (37.8)	
Cancer type [n, (%)]						0.001
BLCA	407 (5.7)	151 (5.4)	119 (7.0)	88 (5.4)	49 (4.8)	
BRCA	1095 (15.3)	434 (15.6)	262 (15.3)	238 (14.6)	161 (15.8)	
CESC	302 (4.2)	110 (4.0)	79 (4.6)	72 (4.4)	41 (4.0)	
CHOL	36 (0.5)	15 (0.5)	5 (0.3)	10 (0.6)	6 (0.6)	
COAD	283 (4.0)	112 (4.0)	79 (4.6)	64 (3.9)	28 (2.8)	
DLBC	48 (0.7)	18 (0.6)	12 (0.7)	12 (0.7)	6 (0.6)	
ESCA	184 (2.6)	61 (2.2)	48 (2.8)	38 (2.3)	37 (3.6)	
HNSC	520 (7.3)	210 (7.6)	121 (7.1)	119 (7.3)	70 (6.9)	
KIRC	533 (7.5)	221 (8.0)	97 (5.7)	146 (8.9)	69 (6.8)	
LAML	173 (2.4)	70 (2.5)	58 (3.4)	29 (1.8)	16 (1.6)	
LIHC	371 (5.2)	127 (4.6)	89 (5.2)	91 (5.6)	64 (6.3)	
LUAD	515 (7.2)	229 (8.3)	114 (6.7)	104 (6.4)	68 (6.7)	
LUSC	501 (7.0)	172 (6.2)	133 (7.8)	107 (6.5)	89 (8.8)	
OV	305 (4.3)	121 (4.4)	59 (3.4)	73 (4.5)	52 (5.1)	
PAAD	178 (2.5)	79 (2.8)	39 (2.3)	36 (2.2)	24 (2.4)	
PRAD	497 (7.0)	213 (7.7)	112 (6.5)	110 (6.7)	62 (6.1)	
READ	93 (1.3)	33 (1.2)	27 (1.6)	19 (1.2)	14 (1.4)	
STAD	415 (5.8)	151 (5.4)	106 (6.2)	108 (6.6)	50 (4.9)	
THCA	505 (7.1)	189 (6.8)	120 (7.0)	120 (7.3)	76 (7.5)	
UCEC	177 (2.5)	59 (2.1)	33 (1.9)	50 (3.1)	35 (3.4)	
Therapeutic efficacy [n, (%)]						0.052
CR/PR	2905 (82.0)	1153 (82.8)	676 (79.2)	676 (84.1)	400 (81.0)	
PD/SD	638 (18.0)	239 (17.2)	177 (20.8)	128 (15.9)	94 (19.0)	

BLCA, bladder urothelial carcinoma; BRCA, breast invasive carcinoma; CESC, cervical squamous cell carcinoma and endocervical adenocarcinoma; CHOL, holangiocarcinoma; DLBC, diffuse large B-cell lymphoma; ESCA, esophageal carcinoma; HNSC, head and neck squamous cell carcinoma; KIRC, kidney renal clear cell carcinoma; LAML, acute myeloid leukemia; LIHC, liver hepatocellular carcinoma; LUAD, lung adenocarcinoma; LUSC, lung squamous cell carcinoma; OV, ovarian serous cystadenocarcinoma; PAAD, pancreatic adenocarcinoma; PRAD, prostate adenocarcinoma; READ, rectum adenocarcinoma; STAD, stomach adenocarcinoma; THCA, thyroid carcinoma; UCEC, uterine corpus endometrial carcinoma; CR, complete response; PR, partial response; SD, stable disease; PD, progressive disease.

**Figure 3:**
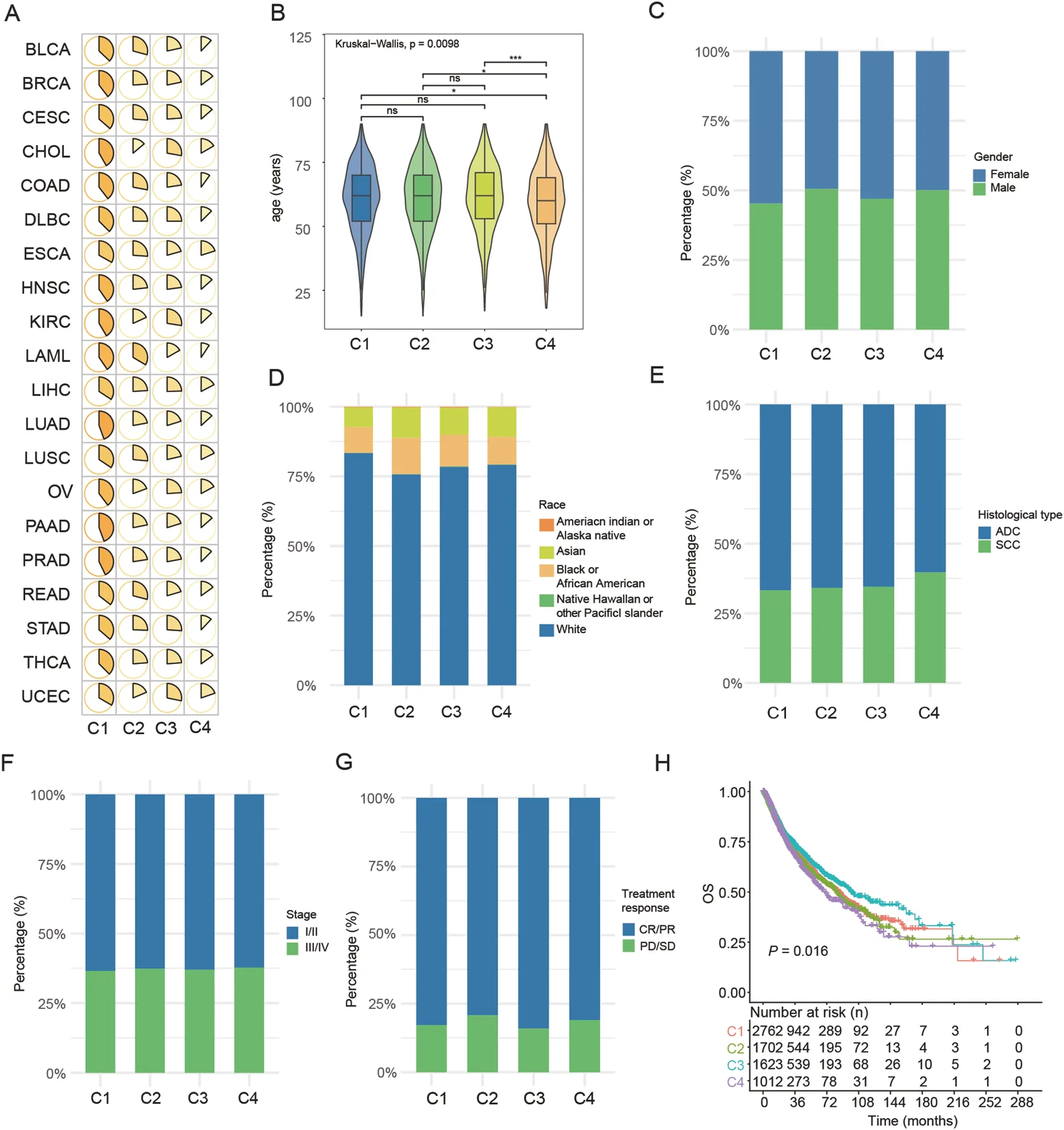
The differences in clinical characteristics, treatment efficacy, and clinical prognosis among the four molecular subtypes. Comparison of distribution of cancer types **(A)**, age **(B)**, gender **(C)**, race **(D)**, histological type **(E)**, stage **(F)**, treatment response **(G)**, and overall survival **(H)** across the four molecular subtypes. * indicates P < 0.05 and *** indicates P < 0.005.

### 3.2 Differences in immune landscape among the four molecular subtypes

Then, we investigated the differences in TIME across distinct subtypes. According to ESTIMATE algorithm, C1 and C3 yielded significantly higher ESTIMATE scores and immune scores compared with C2 and C4 (*P* < 0.001, **Figures 4A, B**). Stromal score was significantly higher in C1 and lower in C2 (*P* < 0.001, **Figure 4C**). Tumor purity was significantly higher in C2 and C4 (*P* < 0.001, **Figure 4D**). These findings demonstrated that C1 and C3 had a larger proportion of immune cell infiltration, whereas C2 and C4 had a higher percentage of tumor cells. Moreover, using CIBERSORT algorithm, we evaluated the estimated proportion of 22 immune cell types. The composition of immune cells across the four subtypes exhibited significant heterogeneity (**Figure 4E**). Specifically, C1 was characterized by high infiltration of resting memory CD4+T cells, resting mast cells, and resting dendritic cells, accompanied by moderate infiltration of CD8+T cells, indicating a moderate immune activity in this subtype. C2 exhibited high infiltration of naive CD4+T cells and naive B cells, coupled with low CD8+T cell infiltration. This suggested that immune cells in this subtype may be largely in a naive state, thereby leading to a weak antitumor immune response. C3 was marked by abundant infiltration of CD8+T cells, activated memory CD4+T cells, follicular helper T cells, and M1 macrophages, together with substantial infiltration of regulatory T cells (Tregs) and M2 macrophages. This implied that patients in this subtype may harbor potent antitumor immunity, yet their TIME was concurrently infiltrated by some immunosuppressive components. C4 exhibited high abundance of M0 macrophages and low abundance of CD8+ T cells, indicating a low antitumor immunity in this subtype.

**Figure 4:**
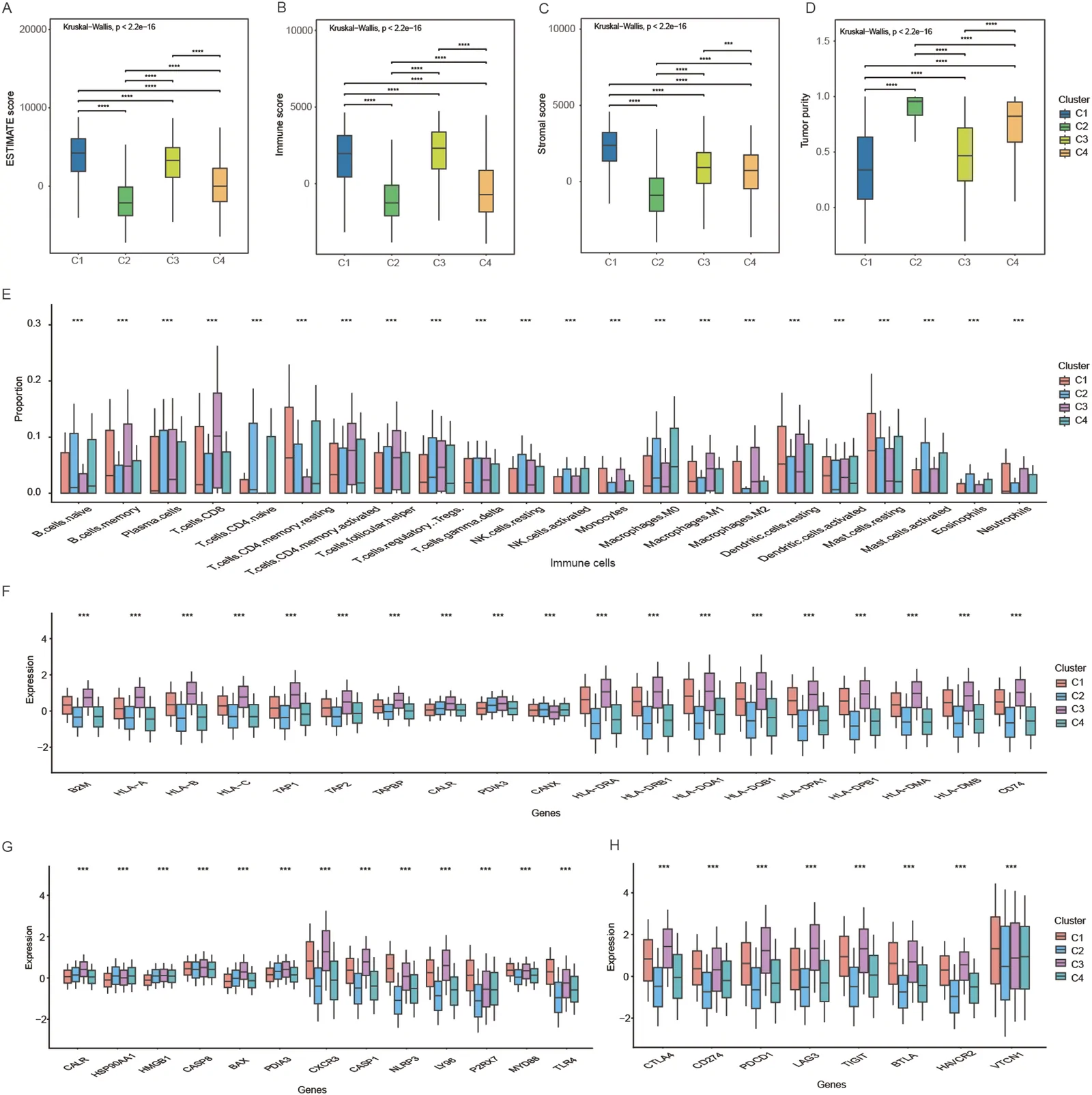
The differences in tumor immune microenvironment among the four molecular subtypes. Comparison of ESTIMATE score **(A)**, immune score **(B)**, stromal score **(C)**, tumor purity **(D)**, the proportion of immune cells **(E)**, as well as the expression of gene related to antigen processing and presentation **(F)**, immune checkpoint **(G)** and immunogenic cell death **(H)** across the four molecular subtypes. *** indicates P < 0.005 and **** indicates P < 0.001.

Furthermore, we compared the differences in the expression of antigen processing and presentation (APP)-related genes, immune checkpoint (ICP) genes and immunogenic cell death (ICD) genes across different subtypes. The results demonstrated that genes involving in MHC class I (e.g. B2M, HLA-A/B/C, TAP1/2, TAPBP) and MHC class II (e.g. HLA-DRA, HLA-DRB1, HLA-DQA1) had significantly higher expression in C1 and C3 relative to C2 and C4 (**Figure 4F**). Similarly, the expression of most ICP (e.g. PD-L1, CTLA4, LAG3 and TIGIT) and ICD (e.g. CALR, CASP1, NLRP3, LY96 and TLR4) genes were significantly higher in C1 and C3 in comparison to C2 and C4 (**Figures 4G, H**). Overall, substantial heterogeneity in the TIME existed across the four molecular subtypes, which may influence patient prognosis and therapeutic responses to immunotherapy.

### 3.3 Developing pan-cancer machine learning classifier based on distinct pathway activity signatures

To improve the clinical translational value of these molecular subtypes, we developed a machine learning classifier. Given that the classifier would be applied to cell line samples, we first performed batch correction on transcriptomic data from the TCGA cohort and cell lines (**Supplementary Figures 2A, B**). Based on GSVA scores of 43 key pathways, the classifier was established in the TCGA training set using the Elastic Net regression algorithm (**Supplementary Table 3**). The classifier exhibited outstanding classification capability, with overall accuracy reaching 96.6% (**Figures 5A, B**). Also, it achieved favorable performance in identifying all molecular subtypes (all sensitivity, specificity, and accuracy >90%, **Figure 5C**). The excellent ability to discriminate distinct subtypes of the classifier was further validated in the TCGA validation set, yielding an overall accuracy of 95.4% (**Figures 5D–F**, **Supplementary Table 4**). Collectively, these results demonstrated that the pan-cancer classifier possessed superior and robust subtyping performance.

**Figure 5:**
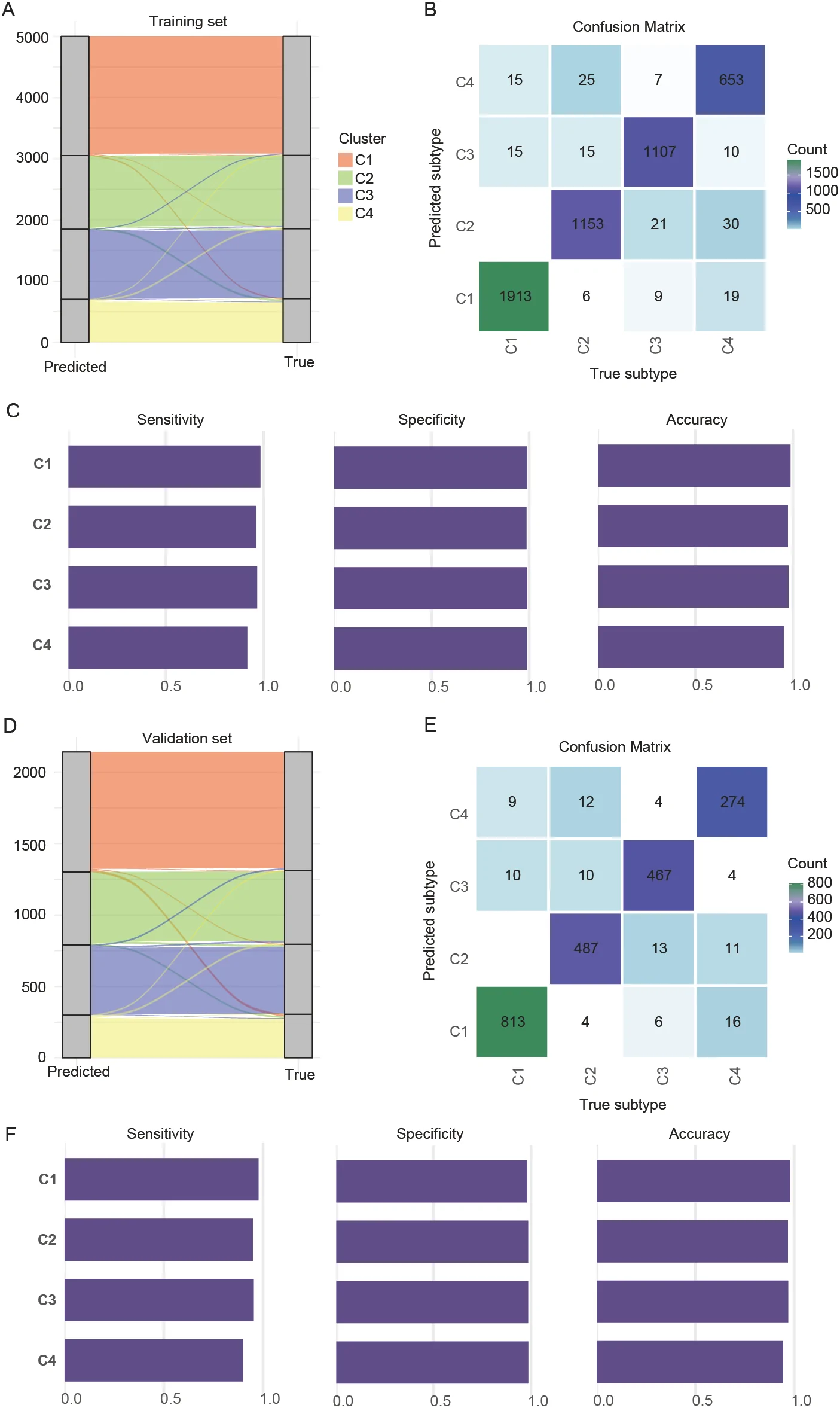
Development of a molecular typing classifier and validation of its classification performance. Sankey diagram **(A)** and confusion matrix **(B)** showing the correspondence between the predicted subtypes and the true subtypes in the training set. **(C)** Sensitivity, specificity, and accuracy of the classifier in identifying each subtype in the training set. Sankey diagram **(D)** and confusion matrix **(E)** showing the correspondence between the predicted subtypes and the true subtypes in the validation set. **(F)** Sensitivity, specificity, and accuracy of the classifier in identifying each subtype in the validation set.

### 3.4 Differences in drug responses among the four molecular subtypes

Furthermore, to explore the differences in drug sensitivity among distinct molecular subtypes, we integrated transcriptomic expression profiles and *in vitro* drug sensitivity datasets of tumor cell lines for subsequent analysis. Considering the inherent biological heterogeneity between cell line models and clinical tumor tissues, we established stringent criteria for cell line subtyping. Only cell lines with a subtype prediction probability greater than 0.9 were assigned to the corresponding subtype. The subtyping results revealed that the pathway activity profiles of each cell line subtype were highly consistent with those of the TCGA cohort (**Supplementary Figure 2C**), which fully demonstrated that this classification system was applicable to cell line samples.

Notably, drug response profiles revealed that C3 exhibited broader sensitivity to most approved anticancer drugs (n=18, 34.6%), investigational compounds (n=21, 25.9%), and experimental chemical probes (n=9, 25%)., whereas other subtypes, particularly C1, showed evident resistance to most compounds (**Supplementary Figure 3**, **Supplementary Table 5**). This consistent response pattern across distinct drugs indicated the existence of a universal mechanism modulating drug response. The marked upregulation of the ABC transporter pathway in the C1 can enhance the drug efflux capacity of tumor cells (19, 20), which may account for its multidrug resistance. In contrast, the significant downregulation of ABCC1 and ABCG2, two key members of the ABC transporter family, in the C3 may underlie its higher drug sensitivity, although no significant intergroup difference was observed in ABCB1 expression (**Supplementary Figure 4**).

In addition, regarding drug classification by targeted pathway, C1 displayed prominent resistance to some compounds targeting cell cycle, including the CDK4/6 inhibitor (CDK4/6i) palbociclib and WEE1 inhibitor adavosertib (**Figure 6A**). Previous studies have delineated the resistance mechanisms of CDK4/6i, such as RB loss, and amplification of E2F and CDK4/6 (21, 22). Furthermore, activation of the PI3K/AKT/mTOR and MAPK signaling pathways has been confirmed to be strongly associated with resistance to CDK4/6i (23, 24). In our study, these two pathways were markedly activated in C1, which may partially explain its insensitivity to palbociclib. Meanwhile, C4 shared the same profile of drug resistance and pathway activation, further corroborating this resistance mechanism. Moreover, C1 was resistant to many compounds targeting DNA replication and genome integrity, including platinum alkylating agents (cisplatin, oxaliplatin), topoisomerase I/II inhibitors (irinotecan, topotecan, mitoxantrone), and the anthracycline antibiotic (epirubicin, **Figure 6B**). This phenomenon may be closely linked to the downregulation of DNA replication and DDR pathways in this cluster. In contrast, C3 showed high sensitivity to these compounds, which may be explained by the marked upregulation of these two pathways. Nevertheless, C2 and C4 also displayed similar upregulation in pathway activity but failed to achieve equivalent drug sensitivity. This finding suggested that the response of these drugs may be modulated by multiple factors, such as the effects of drug efflux mentioned above. Furthermore, C3 showed good responsiveness to venetoclax (**Figure 6C**), which may be correlated with high BCL2 and low BCL-XL expression (**Figures 6D,E**) (25). Furthermore, based on the molecular features of each subtype, three subtypes with poor prognosis were selected to explore whether combination therapy could improve drug sensitivity compared with monotherapy. T24, MDA-MB-453, and BT20 were selected as the cell lines for C1, C2, and C4, respectively, because they were available in the laboratory and exhibited high predictive accuracy. In C1, the ABC transporter and oncogenic signaling pathways were markedly upregulated. Accordingly, we performed drug sensitivity assays using the ABC transporter inhibitor tariquidar combined with the Notch pathway inhibitor DAPT. C2 exhibited prominent upregulation of metabolic and DDR)pathways; therefore, we adopted the glycolysis inhibitor 2-DG in combination with the DDR pathway inhibitor cytarabine for drug sensitivity assays. For C4, DDR and oncogenic signaling pathways were markedly activated, which guided the selection of the DAPT plus cytarabine in the drug sensitivity assays. The results showed that for C1 with up-regulated ABC transporter and oncogenic signaling pathways, C2 with up-regulated metabolic and DDR pathways, and C4 with up-regulated oncogenic signaling and DDR pathways, the optical density (OD) values in the combination therapy groups of pathway-targeted agents were significantly lower than those in the corresponding monotherapy groups (all *P* < 0.05, **Figures 6F–H**). Overall, pathway-based molecular subtypes displayed divergent drug sensitivity profiles, which may provide new insights for precision therapy.

**Figure 6:**
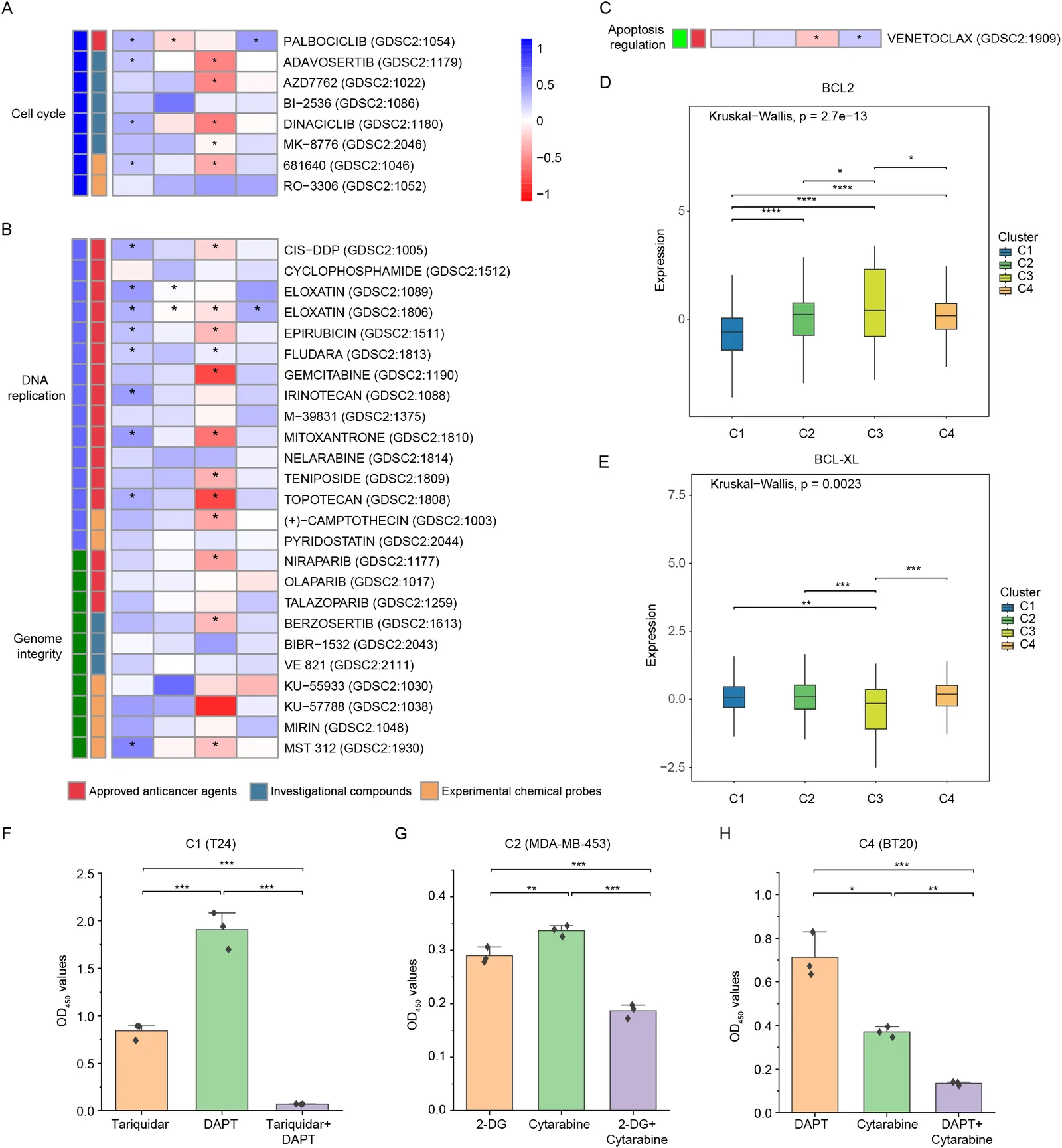
Therapeutic response heterogeneity, underlying molecular mechanisms and subtype-specific combination therapy across four molecular subtypes. Differential responses to drug targeting cell cycle **(A)**, DNA replication and genome integrity **(B)**, and apoptosis regulation **(C)** across four molecular subtypes. Comparison of the expression of BCL2 **(D)** and BCL-XL **(E)** across four molecular subtypes. Comparison of OD values among two monotherapies and their corresponding combination therapy for tariquidar plus DAPT in C1 **(F)**, 2-DG plus cytarabine inC2 **(G)** and DAPT plus cytarabine in C4 **(H)**. * indicates P < 0.05, ** indicates P < 0.01, *** indicates P < 0.005 and **** indicates P < 0.001.

## 4 Discussion

Taken together, the present study categorized pan-cancer specimens into four molecular subtypes based on pathway activity signatures. These subtypes had substantial heterogeneity in core pathway activities, clinical characteristics, therapeutic efficacy, prognostic outcomes, TIME and drug response profiles. These findings elucidated the commonalities and heterogeneities of pan-cancer, providing potential new clues for clinical management of cancers.

Notably, our study successfully identified four molecular subtypes with markedly distinct pathway activation signatures. Specifically, C1 and C4 showed marked upregulation of multiple oncogenic signaling pathways and cellular processes-related pathways. Accumulating evidence suggests that the aberrant activation of oncogenic signaling pathways is closely associated with the onset, progression, and drug resistance of cancer (26, 27). Moreover, it has been reported that the upregulation of cellular processes-related pathways, such as ECM-receptor interaction and focal adhesion, can enhance the adhesion, migration and invasion of tumor cells (28). Meanwhile, the stimulation of oncogenic signaling pathways also promotes epithelial-mesenchymal transition (EMT) (26), indicating that the C1 and C4 may exhibit a highly invasive and metastatic phenotype. Notably, C1 and C4 showed distinct activation patterns of immune and DDR pathways. C4 harbored marked upregulation of the DDR pathways. DNA repair systems maintain genomic integrity and restrain malignant alterations and heterogeneity in tumors, but on the other hand they promote tumor cells survive after radiotherapy and chemotherapy (29). Previous studies have confirmed that aberrant activation of the DDR pathway is closely linked to tumor chemoresistance and immune evasion (30, 31). Moreover, oncogenic signaling pathways extensively interact with the DDR, modulating DNA repair capacity and cellular survival under therapeutic stress (32). In addition, in term of TIME, C4 was characterized by downregulated immune pathways and low infiltration of tumor-killing cells. Collectively, C4 displayed exhibited elevated invasive and metastatic potential, abnormally enhanced DNA repair capacity, and impaired antitumor immunity, which may jointly account for its poorest clinical outcomes.

Unlike C4 with prominent DDR pathway upregulation, C1 showed marked activation of immune pathways, along with moderate CD8+T cell infiltration and elevated expression of ICD/APP-related genes. These findings suggested that although both C1 and C4 possessed high invasive and metastatic potential, the relatively strong antitumor immunity of C1 may be likely to improve patient outcomes. Notably, C3 also showed substantial activation of immune pathways, with highest levels of ICD/APP-related gene expression and CD8+T cell infiltration among all subtypes, reflecting its most potent antitumor immunity. We therefore hypothesized that the favorable clinical outcomes observed in C3 and C1 compared to C2 and C4 may be attributed to their superior antitumor immunity. Concurrently, both C1 and C3 highly expressed ICP genes, and C3 presented high Treg infiltration. It is well established that persistent antigen stimulation drives T cells to express diverse inhibitory receptors (33, 34). Also, effector CD8 T cells rapidly upregulate multiple inhibitory molecules upon activation (35). Based on these observations, C1 and C3 may be “hot tumors”, whereas C2 and C4 may be “cold tumors”. Given the distinct TIME of each subtype, we proposed that the patients in the C1 and C3 may benefit from immunotherapies that reinvigorate dysfunctional T cells, including immune checkpoint inhibitors (ICIs), T-cell engagers, and therapies aimed to diminish Treg cell activity (36), whereas patients in C2 and C4 may benefit from a variety of immunotherapies designed to enhance immune cell infiltration within TME, such as increasing T-cell trafficking to the TME and/or vaccines and adoptive T-cell transfer (37).

Moreover, C2 and C3 exhibited obvious activation of DDR pathways, as well as metabolic pathways, such as glycolysis/gluconeogenesis, oxidative phosphorylation (OXPHOS), fatty acid synthesis (FAS). Abnormal cancer metabolism play a crucial role in tumorigenesis, metastasis, drug resistance, and cancer stem cells (38). As cold tumor, C2 may be prone to adverse biological outcomes driven by metabolic dysregulation. Conversely, C3 harbored abundant immune cell infiltration with TME. Glycolysis is a prerequisite for the proliferation and function of activated CD8+T cells (39, 40), yet large quantities of lactic acid (LA) released by glycolytic tumor cells inhibits CD8+T cell cytotoxicity and facilitates Treg immunosuppression (41). Overall, metabolic pathway activation may exert dual biological effects in the C3.

Of note, the pathway activation signatures and TIME phenotypes derived from TCGA samples, together with drug sensitivity profiles generated from cell lines in each molecular subgroup may provide valuable clues for advancing precision therapy. Specifically, the cell lines in C1 exhibited multidrug resistance, which may be attributed to the upregulation of the ABC transporter pathway and subsequent enhanced drug efflux (19, 20). Accordingly, potential therapeutic benefits for this group could not be elucidated in the current study. However, based on the pathway activation signatures and TIME characteristics of the C1, we hypothesized that this cluster may benefit from combination strategies consisting of drug efflux inhibitors, agents targeting oncogenic signaling pathways and cellular process-related pathways, or ICIs. Moreover, the C2 may derive therapeutic benefit from inhibitors of metabolism pathways, DNA replication, and DDR pathway. The C4 may benefit from agents targeting oncogenic signaling pathways, cellular process-related pathways, DNA replication, and the DDR pathway. For the C3, which featured the best prognosis and broad sensitivity to multiple therapeutic agents, combination regimens incorporating ICIs or agents targeting metabolic pathways, DNA replication, and DDR pathway may be considered to further optimize clinical outcomes (**Figure 7**).

**Figure 7:**
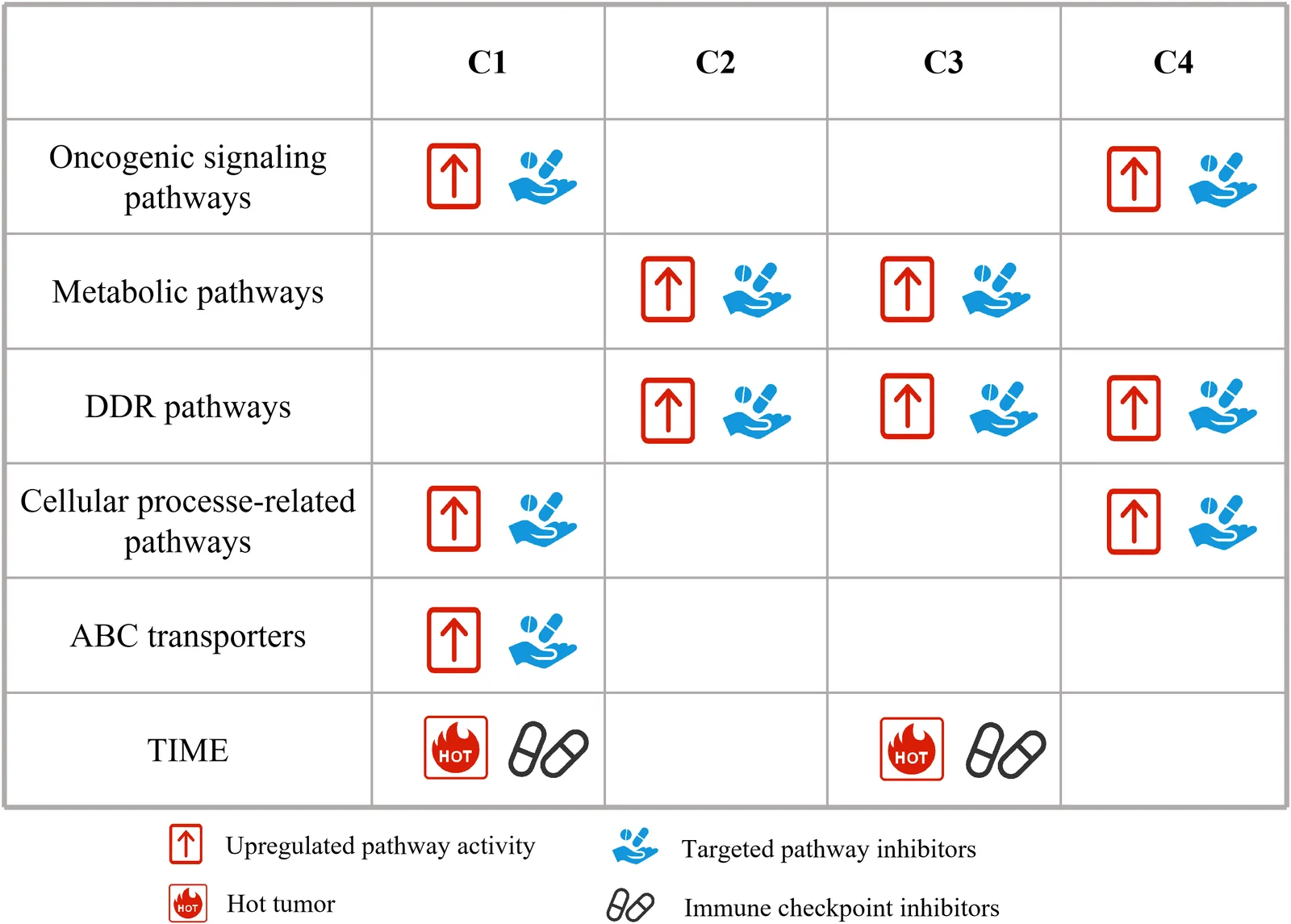
Potential beneficial therapeutic agents for each molecular subgroup inferred from pathway activation signatures and tumor immune microenvironment phenotypes.

There are several limitations in our study. First, due to the lack of an independent external cohort with consistent sequencing platforms and complete prognostic follow-up information, we were unable to perform external validation of prognostic differences among different molecular subtypes. Second, the drug sensitivity data were obtained from cancer cell line models. The findings require further validation using clinical samples or *in vivo* models. Third, our analysis was restricted to transcriptomic data, which limited the comprehensive mechanistic explanations of drug responses. The mechanisms underlying drug responses remain to be thoroughly explored via multi-omics integration and functional experiments in future studies. Given that our findings were limited to retrospective bioinformatic analyses and *in vitro* cellular validations, further pre-clinical and clinical studies are warranted before translating these observations into patient-oriented risk stratification or individualized therapeutic applications.

In summary, this study proposed a method for classifying pan-cancers based on the activity of multiple core pathways. Each molecular subtype exhibited distinct pathway activity aberrations, clinical outcomes, TIME, and drug responses. These findings revealed the heterogeneity and commonality among tumors, which may provide valuable insights for cancer diagnosis and treatment.

## Data Availability

The original contributions presented in the study are included in the article/**Supplementary Material**. Further inquiries can be directed to the corresponding authors.

## Supplementary material

Supplementary Figure 1 Batch effect adjustment of transcriptome data across different cancers. PCA plot of different datasets before **(A)** and after **(B)** batch effect removal. **(C)** Cluster consensus matrices when k=2-10.

Supplementary Figure 2 Batch effect adjustment of transcriptome data form the TCGA cohort and cell lines. PCA plot of different datasets before **(A)** and after **(B)** batch effect removal. **(C)** The heatmap demonstrating the classification accuracy of the molecular subtype classifier validated by similar pathway abnormalities in the cell line cohort.

Supplementary Figure 3 Differential drug response profiles across four molecular subtypes.

Supplementary Figure 4 Comparison of the expression of ABCB1 **(A)**, ABCC1 **(B)** and ABCG2 **(C)** across four molecular subtypes.
